# Rhythmic synchronization and hybrid collective states of globally coupled oscillators

**DOI:** 10.1038/s41598-018-31278-9

**Published:** 2018-08-28

**Authors:** Tian Qiu, Ivan Bonamassa, Stefano Boccaletti, Zonghua Liu, Shuguang Guan

**Affiliations:** 1 Department of Physics, East China Normal University, Shanghai, 200241 China; 2 Institute of Condensed Matter and Material Physics, School of Physics, Peking University, Beijing, 100871 China; 3 Department of Physics, Bar-Ilan University, 52900 Ramat Gan, Israel; 4 CNR-Institute of Complex Systems, Via Madonna del Piano, 10, 50019 Sesto Fiorentino, Florence Italy; 5 The Embassy of Italy in Tel Aviv, 25 Hamered street, 68125 Tel Aviv, Israel

## Abstract

Macroscopic rhythms are often signatures of healthy functioning in living organisms, but they are still poorly understood on their microscopic bases. Globally interacting oscillators with heterogeneous couplings are here considered. Thorough theoretical and numerical analyses indicate the presence of multiple phase transitions between different collective states, with regions of bi-stability. Novel coherent phases are unveiled, and evidence is given of the spontaneous emergence of macroscopic rhythms where oscillators’ phases are always found to be self-organized as in Bellerophon states, i.e. in multiple clusters with quantized values of their average frequencies. Due to their rather unconditional appearance, the circumstance is paved that the Bellerophon states grasp the microscopic essentials behind collective rhythms in more general systems of interacting oscillators.

## Introduction

Rhythmic behaviours are ubiquitous in nature, where we witness events of a rare beauty as, for instance, the acoustic synchrony in cricket’s choruses in the summer or the choreographic dancing of starling flocks in the fall^1,2^. Far from being just a pleasure to our aesthetics, these events reflect actually the correct functioning of living organisms. Brain is a striking example: deterioration or enhancing of neurons’ synchronization may indicate the presence of serious cognitive dysfunctions, like insomnia, epilepsy, or Parkinson’s disease^3–5^.

Synchronization represents a natural scaffold for capturing the microscopic features of these emergent behaviours, and large attention was paid in analyzing the routes towards synchrony in ensembles of dynamical systems^6–8^. In this direction, the Kuramoto model^9^ (and its various generalizations) allowed a wealth of discoveries thanks to its simplicity for a rigorous treatment^9–15^.

Generalized Kuramoto models, in particular, have been the focus of recent research^16^. There, the presence of correlations between the natural frequencies of the oscillators and the coupling strength may lead to first-order like (a.k.a. explosive^15^) phase transitions (PT’s)^17,18^, where the backward (desynchronization from a coherent state) threshold stays fixed, while the forward one (synchronization from incoherence) can be adequately tuned by choosing the system’s parameters (Typically the median of the natural frequency distribution). As soon as the forward transition precedes the backward one, non-stationary rhythmic states called Bellerophon states (B’s) spontaneously emerge^19–23^. Oscillators in B’s are neither phase- nor frequency-locked, but form clusters with quantized averaged frequencies, each one locked to an integer multiple of a principal frequency^23,24^, which can be either odd or even depending on the type of bifurcation (In particular odd B’s appear whenever the incoherent state bifurcates towards a *π*-state, whilst even B’s (also known as oscillating-*π* states) were found at the transition between a traveling wave (TW) and *π*-states (see ref.^24^)).

So far, frequency-coupling correlations in generalized Kuramoto models were designed by following either a dependence of the coupling on the frequencies^16–22^, or an implicit correlation due to a joint distributions in the presence of conformist and contrarian oscillators^23,24^. These arrangements are actually special instances of adding heterogeneity in Kuramoto oscillators’ coupling to the mean field^25–28^, and capture two natural and pervasive features of multi-agent systems, namely modulated strengths of individual responses to the same external stimuli (occurring e.g. in power-grids^29^), and competing interactions^30–32^. In ref.^33^, a detailed analysis of a general coupling form in Kuramoto models was presented, and a number of exotic behavior (such as glassy states and super relaxation) were revealed.

In ref.^23^, a correlation between the oscillator’s natural frequency and the coupling strength was considered, and a frequency term was introduced in the coupling. In ref.^24^, the collective behaviors of a system made of conformist (positively coupled to the mean field) and contrarian (negatively coupled to the mean field) oscillators was studied. Motivated by the fact that real systems in biology^34^ and neuroscience^35^ may actually involve the interplay of such two mechanisms, we here describe the case of a Kuramoto model with an higher order of coupling-heterogeneity, which combines frequency-weighted couplings with positive and negative interactions. By linear stability analysis and mean-field theory, we give analytical predictions of the model’s main thermodynamic properties, which are then validated against extensive numerical simulations. As compared with the results reported in refs^23,24^, we here unveil a novel stationary coherent phase (called strange *π*-state), and give evidence of how generic is the emergence of B’s and of previously unreported rhythmic states (here called hybrid-B’s in light of their microscopic traits). Furthermore, the system undergoes multiple (typically two- or three- stage) PT’s, and features different regimes of bi-stability.

## Results

We start by considering a frequency-weighted Kuramoto model of *N* globally coupled oscillators:

1 $${\dot{\theta }}_{i}={\omega }_{i}+\frac{{\kappa }_{i}|{\omega }_{i}|}{N}\,\sum _{j=1}^{N}\,\sin ({\theta }_{j}-{\theta }_{i}),\,i=1,\ldots ,N,$$

where *θ*_*i*_, *ω*_*i*_, and *κ*_*i*_ are respectively the instantaneous phase of the *i*^th^ oscillator, its natural frequency, and the strength of its coupling to the other oscillators. Here *ω*_*i*_ and *κ*_*i*_ are randomly chosen variables from a joint probability density of the form *G*(*ω*, *κ*) = *g*(*ω*)Γ(*κ*|*ω*), where *g*(*ω*) is the natural frequency distribution – hereafter assumed symmetric and unimodal – and Γ(*κ*|*ω*) is an additional density describing the type of the intrinsic frequency-coupling correlations^36^.

Suppose now that the couplings *κ*_*i*_ take binary (for simplicity, integer) values of opposite signs, i.e. *κ*_1_ < 0 and *κ*_2_ > 0. Oscillators are then grouped into two populations^37,38^: contrarians (opposing the system’s beat) and conformists (attempting to follow the global rhythm). In this scenario, we further specialize on the following three cases:

2 $$I:{{\rm{\Gamma }}}_{1}(\kappa )=(1-p)\,{\delta }_{\kappa ,{\kappa }_{1}}+p\,{\delta }_{\kappa ,{\kappa }_{2}},$$

3 $$II:{{\rm{\Gamma }}}_{2}(\kappa |\,\omega )={\rm{\Theta }}({\omega }_{0}-|\omega |){\delta }_{\kappa ,{\kappa }_{1}}+{\rm{\Theta }}(|\omega |-{\omega }_{0})\,{\delta }_{\kappa ,{\kappa }_{2}},$$

4 $$III:{{\rm{\Gamma }}}_{3}(\kappa |\,\omega )={\rm{\Theta }}(|\omega |-{\omega }_{0})\,{\delta }_{\kappa ,{\kappa }_{1}}+{\rm{\Theta }}({\omega }_{0}-|\omega |)\,{\delta }_{\kappa ,{\kappa }_{2}},$$

where *p* ∈ [0, 1] denotes the proportion of conformist in the model (The fraction *p* ∈ [0, 1] acts as a control parameter of the model; in particular, for *Case II* and *Case III* it is defined respectively as $1-{p}_{2}:\,={\int }_{-{\omega }_{0}}^{{\omega }_{0}}\,g(\omega )d\omega$ and ${p}_{3}:\,={\int }_{-{\omega }_{0}}^{{\omega }_{0}}\,g(\omega )d\omega$, where the subscripts relate to specific cases), ${\rm{\Theta }}(\,\cdot \,)$ is a Heaviside step function, ${\delta }_{(\cdot )}$ is the Kronecker delta. The three densities (2)–(4) reflect three different realistic strategies where contrarians are gradually flipped into conformists (Initially all oscillators are contrarians): in *Case I* randomly chosen contrarians are progressively flipped into conformists, in *Case II* contrarians are ranked according to the magnitude of their natural frequencies |*ω*_*i*_| and then flipped from the largest |*ω*_*i*_| to a given threshold *ω*_0_, and *Case III* is the opposite of *Case II*. As the control parameter *p* is adiabatically tuned, the system (1) typically undergoes a series of PT’s to different coherent states.

The rigorous treatment of model (1) for all the *Cases I*–*III* consists of *i*) performing a linear stability analysis for detecting the thresholds (the forward critical fraction ${p}_{\sigma }^{c}$, *σ* = 1, 2, 3) at which the incoherent state loses stability in the three *Cases I*–*III*, and *ii*) adopting the Kuramotos self-consistent method^9^ for identifying all possible coherent stationary states of the three models, as well as the backward critical thresholds ${p}_{\sigma }^{b}$ at which such coherent states lose their stability. The predicted behaviors are then compared with the numerical solutions obtained integrating directly Eq. (1) (Unless otherwise specified, the following stipulations are chosen: (*i*) the strength of couplings for conformist oscillators is kept fixed to *κ*_2_ = 5; (*ii*) a Lorentzian frequency distribution *g*(*ω*) = *γ*/*π*/[(*ω* − *ω*_0_)^2^ + *γ*^2^] with *γ* = 0.05 is adopted; (*iii*) numerical integrations are performed with a fourth-order Runge-Kutta method with integration time step Δ*t* = 0.01; (*iv*) the initial conditions for the phase variables are randomly taken; (*v*) the typical number of oscillators in the ensemble is *N* = 5 × 10^4^; (*vi*) a sufficiently long time interval (much larger than the oscillation period *T*_*f*_ = 2*π*/Ω_*f*_) is used for the average of the order parameter). In the following, we report the details and results of the linear stability analysis and mean-field theory.

### Linear stability analysis

In the mean-field form, Eq. (1) can be rewritten as:

5 $${\dot{\theta }}_{i}={\omega }_{i}+{\kappa }_{i}|{\omega }_{i}|r\,\sin \,({\rm{\Psi }}-{\theta }_{i}),\,i=1,\ldots ,N,$$

where *r*(*t*) and Ψ(*t*) are amplitude and phase of the Kuramoto order parameter $Z(t)=r(t){e}^{i{\rm{\Psi }}(t)}\,:\,=\frac{1}{N}\,{\sum }_{j=1}^{N}\,{e}^{i{\theta }_{j}}$. Here, *Z*(*t*) can be interpreted as the collective rhythm produced by the whole population of oscillators. The amplitude 0 ≤ *r* ≤ 1 measures the phase coherence in the system and Ψ gives the average phase.

In the thermodynamic limit (*N* → ∞), a density function *ρ*(*θ*, *t*|*ω*, *κ*) can be defined, where *ρ* *dθ* denotes the fraction of oscillators with natural frequency *ω* and coupling strength *κ*, whose phases have values between *θ* and *θ* + *dθ* at time *t*. *ρ* satisfies ${\int }_{[0,2\pi )}\,\rho (\theta ,t|\omega ,\kappa )d\theta =1$ for each *ω*, each *κ*, and all *t*. The evolution of *ρ* is governed by the continuity equation ∂_*t*_*ρ* + ∂_*θ*_(*ρυ*) = 0, where the velocity *υ* is given as *υ* = *ω* + *κ*|*ω*|*r* sin(Ψ − *θ*). Accordingly, the order parameter reads

6 $$Z(t)={\int }_{-\infty }^{\infty }\,{\int }_{-\infty }^{\infty }\,{\int }_{0}^{2\pi }\,{e}^{i\theta }\rho (\theta ,t|\omega ,\kappa )\,{\rm{\Gamma }}(\kappa |\,\omega )g(\omega )d\theta d\kappa d\omega ,$$

where Γ(*κ*|*ω*) could be any of the functions described by Eqs (2–4). The continuity equation can then be rewritten as

7 $$\frac{\partial \rho }{\partial t}=-\,\frac{\partial }{\partial \theta }\,\{\rho [\omega +\kappa |\omega |\,{\int }_{-\infty }^{\infty }\,{\int }_{-\infty }^{\infty }\,{\int }_{0}^{2\pi }\,\sin \,(\theta ^{\prime} -\theta )\rho (\theta ^{\prime} ,t|\omega ^{\prime} ,\kappa ^{\prime} ){\rm{\Gamma }}(\kappa ^{\prime} |\omega ^{\prime} )g(\omega ^{\prime} )d\theta ^{\prime} d\kappa ^{\prime} d\omega ^{\prime} ]\}.$$

For the incoherent state, *ρ*_0_(*θ*, *t*|*ω*, *κ*) = 1/(2*π*). A perturbation

8 $$\rho (\theta ,t|\omega ,\kappa )=\frac{1}{2\pi }+\epsilon \,(\sum _{n=1}^{+\infty }\,{c}_{n}(\omega ,\kappa ,t){e}^{in\theta }+\sum _{n=1}^{+\infty }\,{c}_{n}^{\ast }(\omega ,\kappa ,t){e}^{-in\theta })$$

can be considered, where $\epsilon \ll 1$, and *c*_*n*_ represents the *n*^th^ Fourier coefficient of *ρ*(*θ*, *t*|*ω*, *κ*). Inserting the Fourier expansion (8) into the continuity equation (7), we eventually get the linearized characteristic equations

9 $$\frac{\partial {c}_{1}}{\partial t}=-\,i\omega \cdot {c}_{1}+\frac{\kappa |\omega |}{2}\,{\int }_{-\infty }^{\infty }\,{\int }_{-\infty }^{\infty }\,{c}_{1}(\omega ^{\prime} ,\kappa ^{\prime} ,t){\rm{\Gamma }}(\kappa ^{\prime} |\omega ^{\prime} )g(\omega ^{\prime} )d\kappa ^{\prime} d\omega ^{\prime} $$

and

10 $$\frac{\partial {c}_{n}}{\partial t}=-\,in\omega \cdot {c}_{n},\,n=2,3,\ldots $$

The right-hand side of Eq. (9) defines a linear operator *A* as

11 $$A{c}_{1}=-\,i\omega \cdot {c}_{1}+\frac{\kappa |\omega |}{2}\,{\int }_{-\infty }^{\infty }\,{\int }_{-\infty }^{\infty }\,{c}_{1}(\omega ^{\prime} ,\kappa ^{\prime} ,t){\rm{\Gamma }}(\kappa ^{\prime} |\omega ^{\prime} )g(\omega ^{\prime} )d\kappa ^{\prime} d\omega ^{\prime} ,$$

From Eq. (10), it is obvious that the higher Fourier harmonics are neutrally stable to the perturbation, and the stability of the incoherent state depends on the spectrum of Eq. (9).

The spectrum of *A* has both continuous and discrete sets. Following refs^10,11^, the continuous part of the spectrum is the set {−*iω* : *ω* ∈ Support(*g*)}, which is the whole imaginary axis for a Lorentzian frequency distribution (FD). Therefore, the incoherent state is either unstable or neutrally stable. As for the discrete part of the spectrum, one has to seek solutions of the form *c*_1_(*ω*, *κ*, *t*) = *b*(*ω*, *κ*)*e*^*λt*^, so that the characteristic equation ∂_*t*_*c*_1_ = *A*(*ω*, *κ*)*c*_1_ given in Eq. (11) takes now the form

12 $$\lambda b=-\,i\omega b+\frac{\kappa |\omega |}{2}\,{\int }_{-\infty }^{+\infty }\,{\int }_{-\infty }^{+\infty }\,{\rm{\Gamma }}(\kappa ^{\prime} |\omega ^{\prime} )g(\omega ^{\prime} )b(\omega ^{\prime} ,\kappa ^{\prime} ){\rm{d}}\kappa ^{\prime} {\rm{d}}\omega ^{\prime} .$$

We can then evaluate the latter integral equation self-consistently by defining the auxiliary function

13 $$ {\mathcal B} \equiv {\int }_{-\infty }^{+\infty }\,{\int }_{-\infty }^{+\infty }\,{\rm{\Gamma }}(\kappa ^{\prime} |\omega ^{\prime} )g(\omega ^{\prime} )b(\omega ^{\prime} ,\kappa ^{\prime} ){\rm{d}}\kappa ^{\prime} {\rm{d}}\omega ^{\prime} ,$$

so that Eq. (12) can be solved for *b*(*ω*, *κ*), yielding $b=\frac{\kappa |\omega |}{2}\frac{ {\mathcal B} }{\lambda +i\omega }$ which is well-defined for every $\lambda \in {\mathbb{C}}\backslash \{\,-\,i\omega \}$. Inserting now the expression for *b* into Eq. (13), we are led to the characteristic equation

14 $$2={\int }_{-\infty }^{\infty }\,{\int }_{-\infty }^{\infty }\,\frac{\kappa |\omega |}{(\lambda +i\omega )}{\rm{\Gamma }}(\kappa |\omega )g(\omega )d\kappa d\omega ,\,\lambda \in C\backslash (\,-\,i\omega ),$$

where *λ* is the complex eigenvalue of *A* except for the points in the set {−*iω*}. Notice that Eq. (14) relates implicitly *p* (or *ω*_0_), which serves as the control parameter, with the eigenvalue *λ*. Since the real part of *λ* determines the stability of the incoherent state, we write Eq. (14) into two equations by letting *λ* = *x* + *iy*, i.e.,

15 $$\begin{array}{rcl}1 & = & \frac{1}{2}\,{\int }_{-\infty }^{\infty }\,{\int }_{-\infty }^{\infty }\,\frac{\kappa x}{{x}^{2}+{(\omega -y)}^{2}}|\omega |{\rm{\Gamma }}(\kappa |\omega )g(\omega )d\kappa d\omega ,\\ 0 & = & \frac{1}{2}\,{\int }_{-\infty }^{\infty }\,{\int }_{-\infty }^{\infty }\,\frac{\kappa (\omega -y)}{{x}^{2}+{(\omega -y)}^{2}}|\omega |{\rm{\Gamma }}(\kappa |\omega )g(\omega )d\kappa d\omega .\end{array}$$

Based on Eq. (15), one can determine the stability of the incoherent state, and obtain the critical proportion of conformists (*p*^*c*^) for the forward phase transition.

1. *Case I*. Substituting Γ_1_(*κ*) into Eq. (15) yields
  16 $$1=\frac{(1-{p}_{1}){\kappa }_{1}+{p}_{1}{\kappa }_{2}}{2}\,{\int }_{-\infty }^{\infty }\,\frac{{x}_{1}}{{x}_{1}^{2}+{(\omega -{y}_{1})}^{2}}|\omega |g(\omega )d\omega ,$$
  where the subscript 1 stands for *Case I* (and similar notations have been used for *Case II* and *Case III*). In contrast with the classical Kuramoto model^10,11^, *x*_1_ has not necessarily to be positive, as *κ*_1_ < 0. As *p*_1_ increases, if *x*_1_ changes from negative to positive, the incoherent state will lose its stability. Imposing the critical condition *x*_1_ → 0, one obtains the critical proportion of conformists for the forward PT as
  17 $${p}_{1}^{c}=\frac{2-{\kappa }_{1}\pi {su}{{p}}_{j}\{|{y}_{j}|g({y}_{j})\}}{({\kappa }_{2}-{\kappa }_{1})\pi {su}{{p}}_{j}\{|{y}_{j}|g({y}_{j})\}},$$
  where *y*_*j*_ are determined by Eq. (15) in the limit *x*_1_ → 0, and *y*_*j*_ cannot be zero. Eq. (15) may have more than one root as *x*_1_ → 0. *sup*_*j*_ means that we choose the *j*th root *y*_*j*_ which makes the product |*y*_*j*_|*g*(*y*_*j*_) maximal, so that ${p}_{1}^{c}$ corresponds to the foremost critical point for the onset of synchronization. Specifically, for a Lorentzian FD (Unless otherwise specified, the following stipulations are chosen: (*i*) the strength of couplings for conformist oscillators is kept fixed to *κ*_2_ = 5; (*ii*) a Lorentzian frequency distribution *g(ω*) = *γ/π*/[*(ω* − *ω*_0_)^2^ + *γ*^2^] with *γ* = 0.05 is adopted; (*iii*) numerical integrations are performed with a fourth-order Runge-Kutta method with integration time step Δ*t* = 0.01; (*iv*) the initial conditions for the phase variables are randomly taken; (*v*) the typical number of oscillators in the ensemble is *N* = 5 × 10^4^; (*vi*) a sufficiently long time interval (much larger than the oscillation period *T*_*f*_ = 2*π*/Ω_*f*_) is used for the average of the order parameter), one gets *y*_*j*_ = {0, ±*γ*}. Substituting *y*_*j*_ = ±*γ* into Eq. (17) yields ${p}_{1}^{c}=(4-{\kappa }_{1})/({\kappa }_{2}-{\kappa }_{1})$, which implies that the critical point for the forward PT is uniquely determined by the coupling strengths. In panel (a) of Fig. 1, one sees that these predictions are verified by numerical simulations (at all values of *γ*).

**Figure 1:**
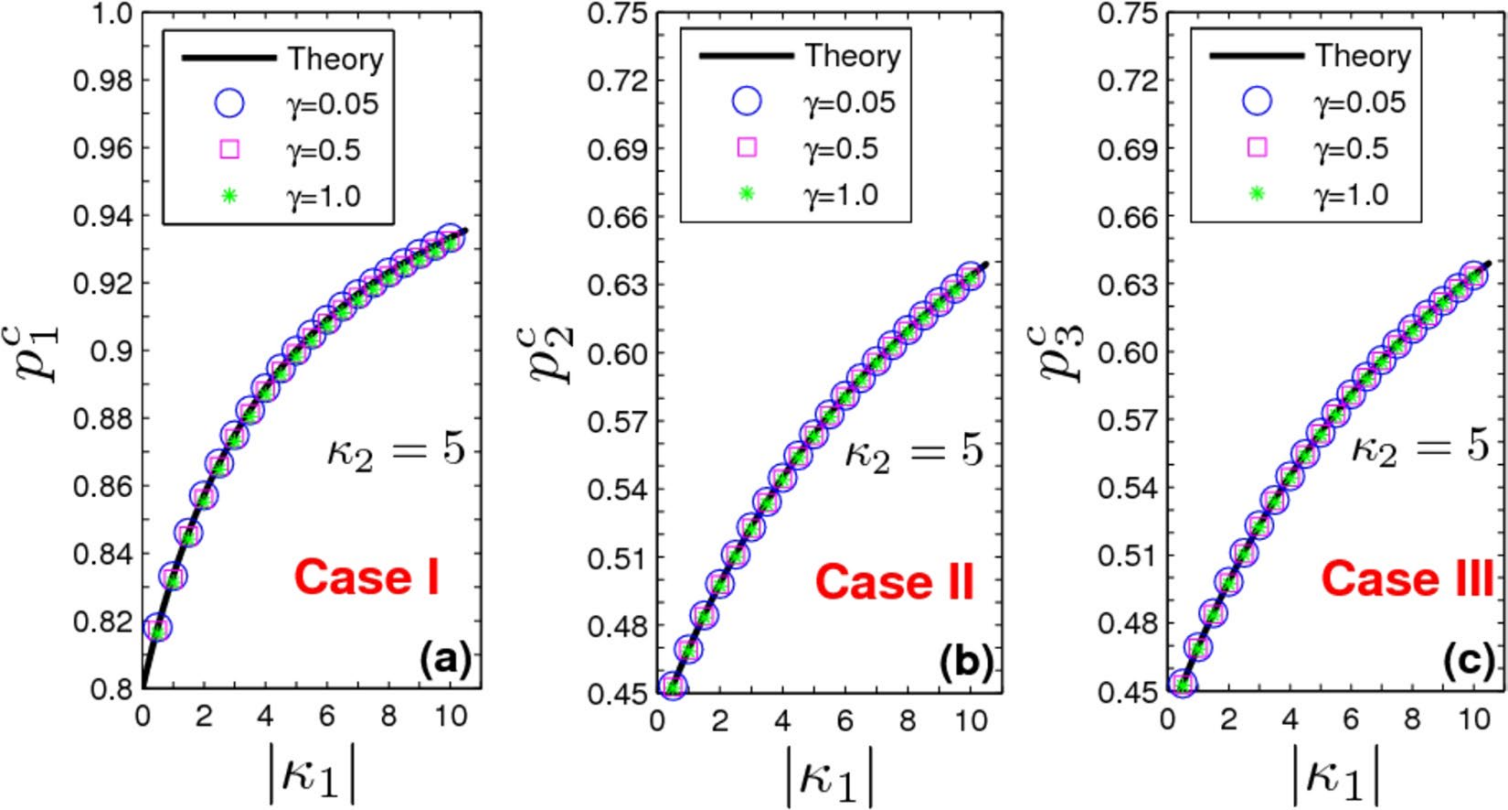
Critical point for the forward phase transition. (**a**) ${p}_{1}^{c}$ *vs*. |*κ*_1_|, (**b**) ${p}_{2}^{c}$ *vs*. |*κ*_1_|, (**c**) ${p}_{3}^{c}$ *vs*. |*κ*_1_|, corresponding to *Cases I*–*III*, respectively. *κ*_2_ = 5.0 in all cases. The numerical results perfectly support the theoretical predictions, at various values of *γ* (reported in the legend).
2. *Case II*. Substituting Γ_2_(*κ*|*ω*) into Eq. (15) yields
  18 $$1=\frac{{\kappa }_{1}}{2}\,{\int }_{-{\omega }_{0}}^{{\omega }_{0}}\,\frac{{x}_{2}|\omega |g(\omega )}{{x}_{2}^{2}+{(\omega -{y}_{2})}^{2}}d\omega +\frac{{\kappa }_{2}}{2}({\int }_{{\omega }_{0}}^{\infty }-{\int }_{-\infty }^{-{\omega }_{0}})\frac{{x}_{2}\omega g(\omega )}{{x}_{2}^{2}+{(\omega -{y}_{2})}^{2}}d\omega .$$
  Also in this case, *x*_2_ is not constrained to be positive. Setting *x*_2_ → 0, one obtains $1={\kappa }_{2}\pi |{y}_{2}^{c}|g({y}_{2}^{c})/2$ and ${y}_{2}^{c}\in (\,-\,\infty ,-\,{\omega }_{0}^{c})\cup ({\omega }_{0}^{c},+\,\infty )$, with ${p}_{2}^{c}$ being determined by Eq. (15), i.e.,
  $$0=P.\,V.\frac{\kappa }{2}\,{\int }_{-\infty }^{\infty }\,{\int }_{-\infty }^{\infty }\,\frac{|\omega |}{\omega -{y}_{2}^{c}}{{\rm{\Gamma }}}_{2}(\kappa |\omega )g(\omega )d\kappa d\omega .$$
  Here, the symbol *P*.*V*. means the Cauchy principal-value integration within the real line. Considering *g*(*ω*) = *g*(−*ω*) and Γ_2_(*κ*|*ω*) = Γ_2_(*κ*| − *ω*), a pair of ${y}_{2}^{c}$ with opposite sign might emerge together. For a Lorentzian FD, one eventually obtains ${y}_{2}^{c}=\pm \,\gamma ({\kappa }_{2}+\sqrt{{\kappa }_{2}^{2}-16})/4$, with ${y}_{2}^{c}\in (\,-\,\infty ,-\,{\omega }_{0}^{c})\cup ({\omega }_{0}^{c},+\,\infty )$, and
  19 $${p}_{2}^{c}=1-\frac{2}{\pi }\,\arctan \,\sqrt{({z}_{2}-{z}_{2}^{\tfrac{-{\kappa }_{1}}{{\kappa }_{2}-{\kappa }_{1}}})/(1+{z}_{2}^{\tfrac{-{\kappa }_{1}}{{\kappa }_{2}-{\kappa }_{1}}})}.$$
  Notice that ${z}_{2}={({y}_{2}^{c}/\gamma )}^{2}$. First, one sees that ${y}_{2}^{c}$ does not exist if *κ*_2_ < 4, suggesting that *x*_2_ → 0 is self-contradictory. Therefore, the real part of *λ*_2_ is either positive or negative when *κ*_2_ < 4. Obviously, *x*_2_ > 0 is physically unreasonable. Therefore, *x*_2_ is supposed to be negative in this case, which implies that the coherent state will not emerge no matter how large the proportion of conformist is. Then, when *κ*_2_ > 4, one can obtain the critical proportion of conformists for forward PT, which is reported in panel (b) of Fig. 1.
3. *Case III*. Substituting Γ_3_(*κ*|*ω*) into Eq. (15), and setting *x*_3_ → 0, yields $1={\kappa }_{2}\pi |{y}_{3}^{c}|g({y}_{3}^{c})/2$, and ${y}_{3}^{c}\in (\,-\,{\omega }_{0}^{c},{\omega }_{0}^{c})$. Taking again a Lorentzian FD, when *κ*_2_ < 4 PT is impossible because *x*_3_ is always negative. When *κ*_2_ > 4, one obtains ${y}_{3}^{c}=\pm \,\gamma ({\kappa }_{2}-\sqrt{{\kappa }_{2}^{2}-16})/4$, ${y}_{3}^{c}\in (\,-\,{\omega }_{0}^{c},{\omega }_{0}^{c})$, and

20 $${p}_{3}^{c}=\frac{2}{\pi }\,\arctan \,\sqrt{({z}_{3}+{z}_{3}^{\tfrac{{\kappa }_{2}}{{\kappa }_{2}-{\kappa }_{1}}})/(1-{z}_{3}^{\tfrac{{\kappa }_{2}}{{\kappa }_{2}-{\kappa }_{1}}})}$$

in the limit *x*_3_ → 0, where ${z}_{3}={({y}_{3}^{c}/\gamma )}^{2}$. Remarkably, *Case II* and *Case III* have the same critical point for the forward PT, i.e., ${p}_{2}^{c}={p}_{3}^{c}$. This can be proved briefly as follows. Because of *z*_2_*z*_3_ = 1, one has

$$f(z)=\sqrt{\frac{{z}_{2}-{z}_{2}^{\frac{-{\kappa }_{1}}{{\kappa }_{2}-{\kappa }_{1}}}}{1+{z}_{2}^{\frac{-{\kappa }_{1}}{{\kappa }_{2}-{\kappa }_{1}}}}}={(\frac{{z}_{3}+{z}_{3}^{\frac{{\kappa }_{2}}{{\kappa }_{2}-{\kappa }_{1}}}}{1-{z}_{3}^{\frac{{\kappa }_{2}}{{\kappa }_{2}-{\kappa }_{1}}}})}^{-1/2}.$$

Now, since $\arctan \,f(z)+\arctan \,\tfrac{1}{f(z)}=\arctan \,\tfrac{f(z)+\tfrac{1}{f(z)}}{1-f(z)\tfrac{1}{f(z)}}=\tfrac{\pi }{2}$, one straightforwardly obtains ${p}_{2}^{c}={p}_{3}^{c}$. The theoretical and numerical results are shown in panel (c) of Fig. 1.

### Mean-field theory

In order to unveil all possible coherent states in the system (as the proportion of conformists *p* increases), one has to rely on self-consistence analysis. For stationary coherent state, the mean-field phase Ψ rotates uniformly with frequency Ω, i.e., Ψ(*t*) = Ω*t* + Ψ(0). Without loss of generality, one can set Ψ = 0 after an appropriate time shift. In the rotating frame with frequency Ω, the phase difference *ϕ*_*i*_ = *θ*_*i*_ − Ψ is introduced, and the model can be written as

21 $${\dot{\varphi }}_{i}={\omega }_{i}-{\rm{\Omega }}-{\kappa }_{i}|{\omega }_{i}|r\,\sin \,{\varphi }_{i},\,i=1,\ldots ,N$$

in the rotating frame. In the Kuramoto model, the mean field may fluctuate at a rhythm different from the ensemble average (or the mode average) of the natural frequencies of the oscillators, especially when asymmetry in the coupling strengths is present^39^. Notice that there is no guarantee that Ω vanishes in Eq. (21) because of the asymmetry in the efficient coupling parameters *κ*_*i*_|*ω*_*i*_|. Eq. (21) should be discussed for the two distinct populations: the phase-locked and the drifting oscillators. On the one hand, when |*ω*_*i*_ − Ω| ≤ |*κ*_*i*_*ω*_*i*_*r*|, Eq. (21) has a fixed point (${\dot{\varphi }}_{i}=0$) solution, given by sin *ϕ*_*i*_ = (*ω*_*i*_ − Ω)/(*κ*_*i*_|*ω*_*i*_|*r*), corresponding to the phase-locked oscillators entrained by the mean-field. On the other hand, for the drifting oscillators, |*ω*_*i*_ − Ω| > |*κ*_*i*_*ω*_*i*_*r*|. The order parameter in Eq. (6) can be rewritten as

22 $$r=\frac{1}{N}\,\sum _{j=1}^{N}\,{e}^{i({\theta }_{j}-{\rm{\Psi }})}=\frac{1}{N}\,\sum _{j=1}^{N}\,{e}^{i{\varphi }_{j}}=\frac{1}{N}\,\sum _{j=1}^{N}\,{e}^{i{\varphi }_{j}}H(1-|\frac{{\omega }_{j}-{\rm{\Omega }}}{{\kappa }_{j}{\omega }_{j}r}|)+\frac{1}{N}\,\sum _{j=1}^{N}\,{e}^{i{\varphi }_{j}}H(|\frac{{\omega }_{j}-{\rm{\Omega }}}{{\kappa }_{j}{\omega }_{j}r}|-1).$$

When replacing summation by integration, the contribution of the phase-locked oscillators to *r* reads

23 $$\begin{array}{l}\pm \,{\iint }_{{{\mathbb{R}}}^{2}}\,\sqrt{1-{(\frac{\omega -{\rm{\Omega }}}{\kappa \omega r})}^{2}}{\rm{\Gamma }}(\kappa |\omega )g(\omega )H\,(1-|\frac{\omega -{\rm{\Omega }}}{\kappa \omega r}|)\,d\kappa d\omega \\ \,+\,i\,{\iint }_{{{\mathbb{R}}}^{2}}\,\frac{\omega -{\rm{\Omega }}}{\kappa |\omega |r}{\rm{\Gamma }}(\kappa |\omega )g(\omega )H\,(1-|\frac{\omega -{\rm{\Omega }}}{\kappa \omega r}|)d\kappa d\omega ,\end{array}$$

where conformists take the positive sign and contrarians take the negative sign in the first integral. This is because, in a stationary coherent state, conformists attempt to follow the global rhythm of the system (and therefore cos *ϕ*_*i*_ > 0 is always the case), whereas contrarians try to oppose the system’s mean-field featuring cos *ϕ*_*i*_ < 0.

In contrast to phase-locked oscillators, the drifting oscillators cannot be entrained by the mean-field. Self-consistently, they form a stationary distribution on the circle^11^, i.e., ∂*ρ*/∂*t* = 0, and the constant value of the order parameter must be consistent with that implied by Eq. (22). Then, the distribution of the drifting oscillators in the rotating frame is given by

$$\rho (\varphi |\omega ,\kappa )=\frac{\sqrt{{(\omega -{\rm{\Omega }})}^{2}-{(\kappa \omega r)}^{2}}}{2\pi |\omega -{\rm{\Omega }}-\kappa |\omega |r\,\sin \,\varphi |}.$$

It is then easy to obtain that, for drifting oscillators, $\langle \cos \,\varphi \rangle ={\int }_{0}^{2\pi }\,\rho (\varphi |\omega ,\kappa )\,\cos \,\varphi \,d\varphi =0$, and

$$\langle \sin \,\varphi \rangle ={\int }_{0}^{2\pi }\,\rho (\varphi ,\omega ,\kappa )\,\sin \,\varphi \,d\varphi =\frac{\omega -{\rm{\Omega }}}{\kappa |\omega |r}\,[1-\sqrt{1-{(\frac{\kappa \omega r}{\omega -{\rm{\Omega }}})}^{2}}].$$

The drifting oscillators have no contributions to the real part of *r*. However, their contributions to the imaginary part of *r* should not be neglected. The closed form of self-consistence equations for the real and imaginary parts of *r* are

24 $$r={\int }_{-\infty }^{\infty }\,{\int }_{-\infty }^{\infty }\pm \sqrt{1-{(\frac{\omega -{\rm{\Omega }}}{\kappa \omega r})}^{2}}{\rm{\Gamma }}(\kappa |\omega )g(\omega )H\,(1-|\frac{\omega -{\rm{\Omega }}}{\kappa \omega r}|)\,d\kappa d\omega ,$$

and

25 $$\begin{array}{rcl}0 & = & {\int }_{-\infty }^{\infty }\,{\int }_{-\infty }^{\infty }\,\frac{\omega -{\rm{\Omega }}}{\kappa |\omega |r}\,{\rm{\Gamma }}(\kappa |\omega )g(\omega )H\,(1-|\frac{\omega -{\rm{\Omega }}}{\kappa \omega r}|)\,d\kappa d\omega \\  &  & +\,{\int }_{-\infty }^{\infty }\,{\int }_{-\infty }^{\infty }\,\frac{\omega -{\rm{\Omega }}}{\kappa |\omega |r}\,[1-\sqrt{1-{(\frac{\kappa \omega r}{\omega -{\rm{\Omega }}})}^{2}}]\,{\rm{\Gamma }}(\kappa |\omega )g(\omega )H\,(|\frac{\omega -{\rm{\Omega }}}{\kappa \omega r}|-1)\,d\kappa d\omega .\end{array}$$

Taken together, Eqs (24) and (25) provide a closed equation for the dependence of the magnitude *r* and the frequency of the mean-field Ω on *p*. We notice that Ω = 0 is always a trivial solution of Eq. (25), corresponding to the *π*-state reported in refs^37,38^. In this state, the conformist and contrarian oscillators converge to a partially synchronized state where they both satisfy a stationary distribution of phases, and the phase difference between these two clusters is always *δ* = *π*. Since Ω = 0 may not be the only solution, there could be more than one value for Ω that satisfies the phase balance equation. ${\rm{\Omega }}\ne 0$ corresponds to the travelling wave (TW) state, in which the two clusters always maintain a constant separation $\delta \ne \pi$, and rotate with the same frequency along the unit circle, i.e., they are relatively static with each other.

**Figure 2:**
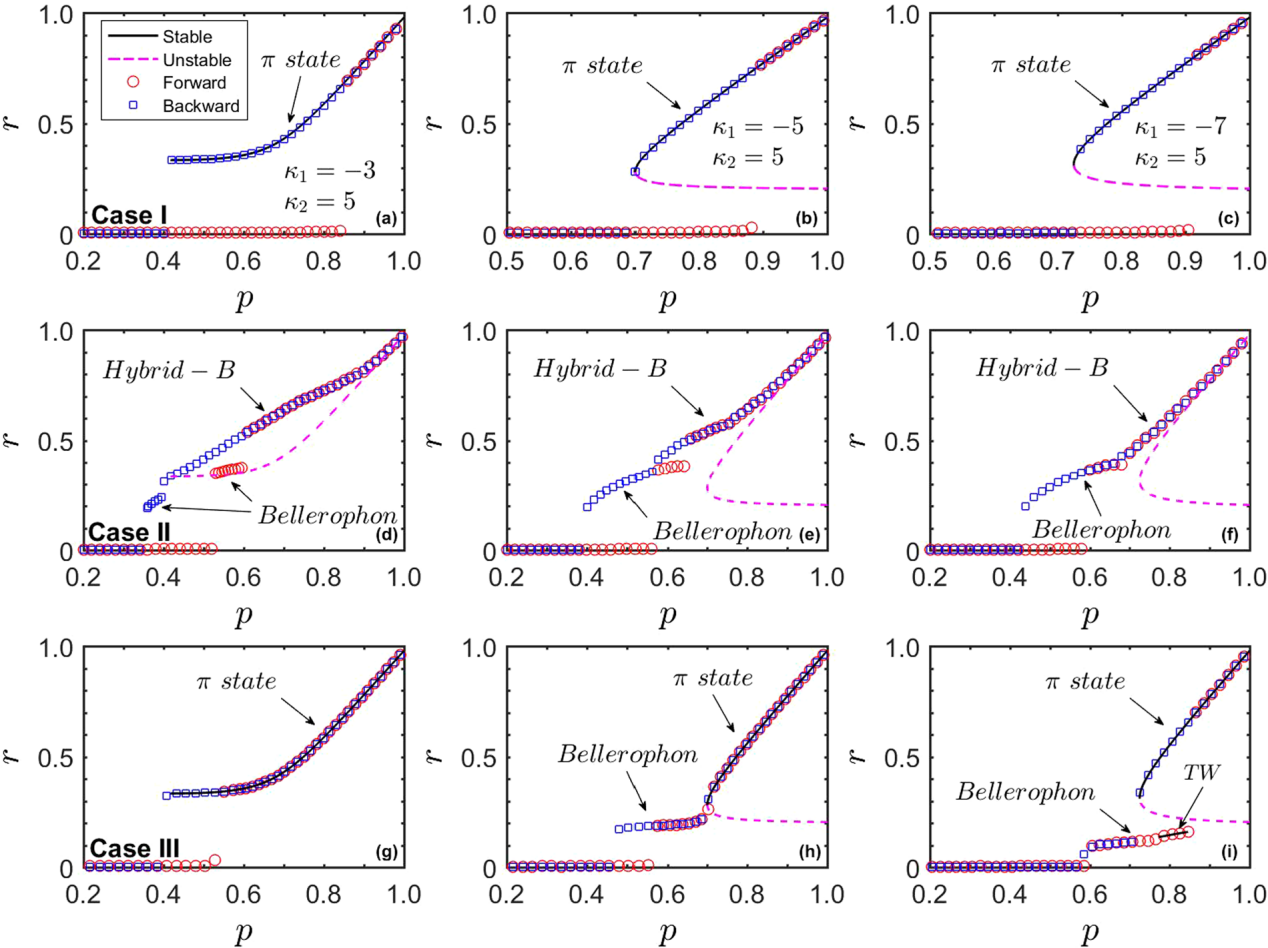
Typical synchronization scenarios in Eq. (1) as the proportion of conformists *p* increases. Lorentzian FD with *γ* = 0.05. From top to bottom, the three rows correspond to *Cases I*–*III*, respectively, while from left to right, the three columns correspond to *Q* < 1, *Q* = 1, and *Q* > 1, respectively (*Q* = |*κ*_1_|/*κ*_2_). Both the forward (red circles) and the backward (blue squares) transitions are studied in an adiabatic way, and the black (dashed pinkish red) curves correspond to theoretical predictions of the stable (unstable) stationary coherent states, including the *π*-state and the TW states. Other typical non-stationary coherent states, such as the strange *π*-state, the B-state, and the hybrid-B state, can also be observed in broad parameter ranges.
  For the case of *α*_1,2_ < 1, Ω_1_ is not supposed to be 0, the solution of Eqs (26) and (27) can be solved numerically, corresponding to the TW state. Particularly, in the limit case *r* → 0^+^, one can obtain ${p}_{1}^{c}$ again, which is exactly the same as Eq. (17):
  30 $${p}_{1}^{c}=\frac{2-{\kappa }_{1}\pi |{{\rm{\Omega }}}_{1}^{c}|g({{\rm{\Omega }}}_{1}^{c})}{({\kappa }_{2}-{\kappa }_{1})\pi |{{\rm{\Omega }}}_{1}^{c}|g({{\rm{\Omega }}}_{1}^{c})},$$
  where ${{\rm{\Omega }}}_{1}^{c}$ is the critical mean-field frequency. By Taylor expansion of Eq. (27), we find that ${{\rm{\Omega }}}_{1}^{c}$ satisfies the following balance equation,
  31 $$0=P.\,V.\,{\int }_{-\infty }^{\infty }\,\frac{|\omega |g(\omega )}{\omega -{{\rm{\Omega }}}_{1}^{c}}\,d\omega .$$
  From Eq. (30), we know that ${{\rm{\Omega }}}_{1}^{c}\ne 0$ when the incoherent state loses its stability, i.e., a stationary TW state will emerge when the system get synchronized. Although a linear stability analysis to this TW state is hard to be performed, its stability can still be studied through numerical simulations. It is found that the TW state predicted by the mean-field theory turns out to be unstable. Taking a Lorentzian FD, Eqs (26) and (27) can be simplified to
  32 $$\begin{array}{rcl}r & = & -\frac{(1-{p}_{1})\gamma }{\pi }\,{\int }_{{\kappa }_{1}r}^{-{\kappa }_{1}r}\,\sqrt{1-{(\frac{x}{{\kappa }_{1}r})}^{2}}\frac{|{{\rm{\Omega }}}_{1}|}{{{\rm{\Omega }}}_{1}^{2}+{\gamma }^{2}{(1-x)}^{2}}\,dx\\  &  & +\,\frac{{p}_{1}\gamma }{\pi }\,{\int }_{-{\kappa }_{2}r}^{{\kappa }_{2}r}\,\sqrt{1-{(\frac{x}{{\kappa }_{2}r})}^{2}}\frac{|{{\rm{\Omega }}}_{1}|}{{{\rm{\Omega }}}_{1}^{2}+{\gamma }^{2}{(1-x)}^{2}}\,dx,\end{array}$$
  33 $$\begin{array}{rcl}0 & = & [\frac{(1-{p}_{1})}{{\kappa }_{1}}\,{\int }_{{\kappa }_{1}r}^{-{\kappa }_{1}r}+\frac{{p}_{1}}{{\kappa }_{2}}\,{\int }_{-{\kappa }_{2}r}^{{\kappa }_{2}r}]\,\frac{x}{{{\rm{\Omega }}}_{1}^{2}+{\gamma }^{2}{(1-x)}^{2}}\,dx\\  &  & +\,\frac{(1-{p}_{1})}{{\kappa }_{1}}\,({\int }_{-\infty }^{{\kappa }_{1}r}+{\int }_{-{\kappa }_{1}r}^{1}-{\int }_{1}^{\infty })\,[1-\sqrt{1-{(\frac{{\kappa }_{1}r}{x})}^{2}}]\,\frac{x}{{{\rm{\Omega }}}_{1}^{2}+{\gamma }^{2}{(1-x)}^{2}}\,dx\\  &  & +\,\frac{{p}_{1}}{{\kappa }_{2}}\,({\int }_{-\infty }^{-{\kappa }_{2}r}+{\int }_{{\kappa }_{2}r}^{1}-{\int }_{1}^{\infty })\,[1-\sqrt{1-{(\frac{{\kappa }_{2}r}{x})}^{2}}]\,\frac{x}{{{\rm{\Omega }}}_{1}^{2}+{\gamma }^{2}{(1-x)}^{2}}\,dx.\end{array}$$
  Numerical simulations suggest that this bifurcation is supercritical and unstable. Therefore, above ${p}_{1}^{c}$ the incoherent state (*α*_1,2_ < 0) loses its stability and the system jumps to *π*-state (*α*_1,2_ > 0) predicted by Eq. (29) because the TW state is unstable, i.e., a first-order synchronization transition takes place.
  In our numerical studies, it is found that in the backward PT, however, the *π*-state does not always return to incoherence directly as shown in Fig. 2(a–c), and rather bifurcates continuously (for small enough *κ*_1_) towards a novel stationary state, here called the strange *π*-state. For example, in Fig. 3(a), we plot the phase diagram for *κ*_1_ = −2 and *κ*_2_ = 5. As *p*_1_ decreases, when the *π*-state loses stability, instead of returning back to incoherence state directly, the system bifurcates to the strange *π*-state. In Fig. 3(b), we show the microscopic characterization of this state. It is found that in this state, the contrarian clusters have desynchronized, while the conformist clusters still maintain complete coherence. The contrarians maximize their distance from the conformists’ clusters, resulting in an averaged phase difference of *π*. Physically, this can be understood as follows. When |*κ*_1_| is significantly smaller than *κ*_2_, contrarians are less affected by the mean field, and thus are easier to desynchronize as *p* decreases. Furthermore, as Ω_1_ = 0, we can predict this kind of state through mean-field method. Now, one should remove the contribution of contrarian clusters in Eq. (26), and get ${p}_{1}={r}^{2}/\sqrt{{r}^{2}-{(1/{\kappa }_{2})}^{2}}$. As shown in Fig. 3(a), the theoretical predictions agree perfectly with the numerical results.

1. *Case I*.
  Substituting Γ_1_(*κ*) into Eqs (24) and (25) yields
  26 $$\begin{array}{rcl}r & = & -(1-{p}_{1})\,{\int }_{-\infty }^{\infty }\,\sqrt{1-{(\frac{\omega -{{\rm{\Omega }}}_{1}}{{\kappa }_{1}\omega r})}^{2}}g(\omega )H\,(1-|\frac{\omega -{{\rm{\Omega }}}_{1}}{{\kappa }_{1}\omega r}|)\,d\omega \\  &  & +\,{p}_{1}\,{\int }_{-\infty }^{\infty }\,\sqrt{1-{(\frac{\omega -{{\rm{\Omega }}}_{1}}{{\kappa }_{2}\omega r})}^{2}}g(\omega )H\,(1-|\frac{\omega -{{\rm{\Omega }}}_{1}}{{\kappa }_{2}\omega r}|)\,d\omega ,\end{array}$$
  27 $$\begin{array}{rcl}0 & = & (1-{p}_{1})\,{\int }_{-\infty }^{\infty }\,\frac{\omega -{{\rm{\Omega }}}_{1}}{{\kappa }_{1}|\omega |r}g(\omega )H\,(1-|\frac{\omega -{{\rm{\Omega }}}_{1}}{{\kappa }_{1}\omega r}|)\,d\omega \\  &  & +\,{p}_{1}\,{\int }_{-\infty }^{\infty }\,\frac{\omega -{{\rm{\Omega }}}_{1}}{{\kappa }_{2}|\omega |r}\,g(\omega )H(1-|\frac{\omega -{\Omega }_{1}}{{\kappa }_{2}\omega r}|)\,d\omega \\  &  & +\,(1-{p}_{1})\,{\int }_{-\infty }^{\infty }\,\frac{\omega -{{\rm{\Omega }}}_{1}}{{\kappa }_{1}|\omega |r}\,[1-\sqrt{1-{(\frac{{\kappa }_{1}\omega r}{\omega -{{\rm{\Omega }}}_{1}})}^{2}}]\,g(\omega )H\,(|\frac{\omega -{{\rm{\Omega }}}_{1}}{{\kappa }_{1}\omega r}|-1)\,d\omega \\  &  & +\,{p}_{1}\,{\int }_{-\infty }^{\infty }\,\frac{\omega -{{\rm{\Omega }}}_{1}}{{\kappa }_{2}|\omega |r}\,[1-\sqrt{1-{(\frac{{\kappa }_{2}\omega r}{\omega -{{\rm{\Omega }}}_{1}})}^{2}}]\,g(\omega )H\,(|\frac{\omega -{{\rm{\Omega }}}_{1}}{{\kappa }_{2}\omega r}|-1)\,d\omega ,\end{array}$$
  where subscript 1 in *κ*_1_ and Ω_1_ denotes *Case I* (and similar notations have been used for *Case II* and *Case III*).
  Defining *α*_1_ = *κ*_1_*r*, *α*_2_ = *κ*_2_*r* and *x* = (*ω* − Ω_1_)/*ω*, ${{\rm{\Omega }}}_{1}\ne 0$, Eq. (26) can be expressed as
  28 $$\begin{array}{rcl}r & = & -(1-{p}_{1})\,{\int }_{-\infty }^{\infty }\,\sqrt{1-{(\frac{x}{{\alpha }_{1}})}^{2}}g\,(\frac{{{\rm{\Omega }}}_{1}}{1-x})\,\frac{|{{\rm{\Omega }}}_{1}|}{{(1-x)}^{2}}\,H\,(\,-\,{\alpha }_{1}-|x|)\,dx\\  &  & +\,{p}_{1}\,{\int }_{-\infty }^{\infty }\,\sqrt{1-{(\frac{x}{{\alpha }_{2}})}^{2}}g\,(\frac{{{\rm{\Omega }}}_{1}}{1-x})\,\frac{|{{\rm{\Omega }}}_{1}|}{{(1-x)}^{2}}\,H\,({\alpha }_{2}-|x|)\,dx.\end{array}$$
  For partially synchronous state, *α*_1,2_ > 1. To avoid divergency of Eq. (28), the only choice is Ω_1_ = 0, and Eq. (26) is reduced as
  29 $${p}_{1}=\frac{{r}^{2}+\sqrt{{r}^{2}-{(1/{\kappa }_{1})}^{2}}}{\sqrt{{r}^{2}-{(1/{\kappa }_{1})}^{2}}+\sqrt{{r}^{2}-{(1/{\kappa }_{2})}^{2}}}.$$
  From Eq. (29), one can extract the critical proportion of conformists for backward PT as well as the *π*-state^37,38^ theoretically. Interestingly, this solution is independent of the specific form of *g*(*ω*) as long as *g*(*ω*) is symmetric and centered at 0, which is like the case of two-cluster synchronous state in ref.^17^. The critical proportion of conformists for backward PT (${p}_{1}^{b}$) is the minimum *p*_1_ which satisfies Eq. (29), thus ${p}_{1}^{b}$ can be determined by setting *dp*_1_/*dr* = 0 in Eq. (29). When |*κ*_1_| = *κ*_2_ = *κ*, ${p}_{1}^{b}=(2+\kappa )/2\kappa$ and ${r}_{1}^{b}=\sqrt{2}/\kappa$. When |*κ*_1_| < *κ*_2_, ${p}_{1}^{b}=2/{\kappa }_{2}$ and ${r}_{1}^{b}=\sqrt{2}/{\kappa }_{2}$. When |*κ*_1_| > *κ*_2_, the equation for ${p}_{1}^{b}$ turns out to be tedious so we do not show the exact results here.
  In Fig. 2(a–c), we have plotted the phase diagram for typical parameters in *Case I*, as well as the solutions of Eq. (29), which perfectly coincide with the numerical results. In *Case I*, the typical coherent state is the *π*-state. In this state, there are four coherent clusters in the system, including a pair of conformist clusters and a pair of contrarian clusters. They are all static and their phases are locked with zero average speed, thus the order parameter is a fixed point on the complex plane. Moreover, the average phase of all conformists and that of all contrarians maintain a constant difference of *π*. During the backward transition, as *p*_1_ decreases, contrarian clusters first begin to desynchronize, while conformist clusters still keep synchronized. Only when *p*_1_ becomes small enough, the conformist clusters begin to desynchronize and the system finally returns back to the incoherent state.

**Figure 3:**
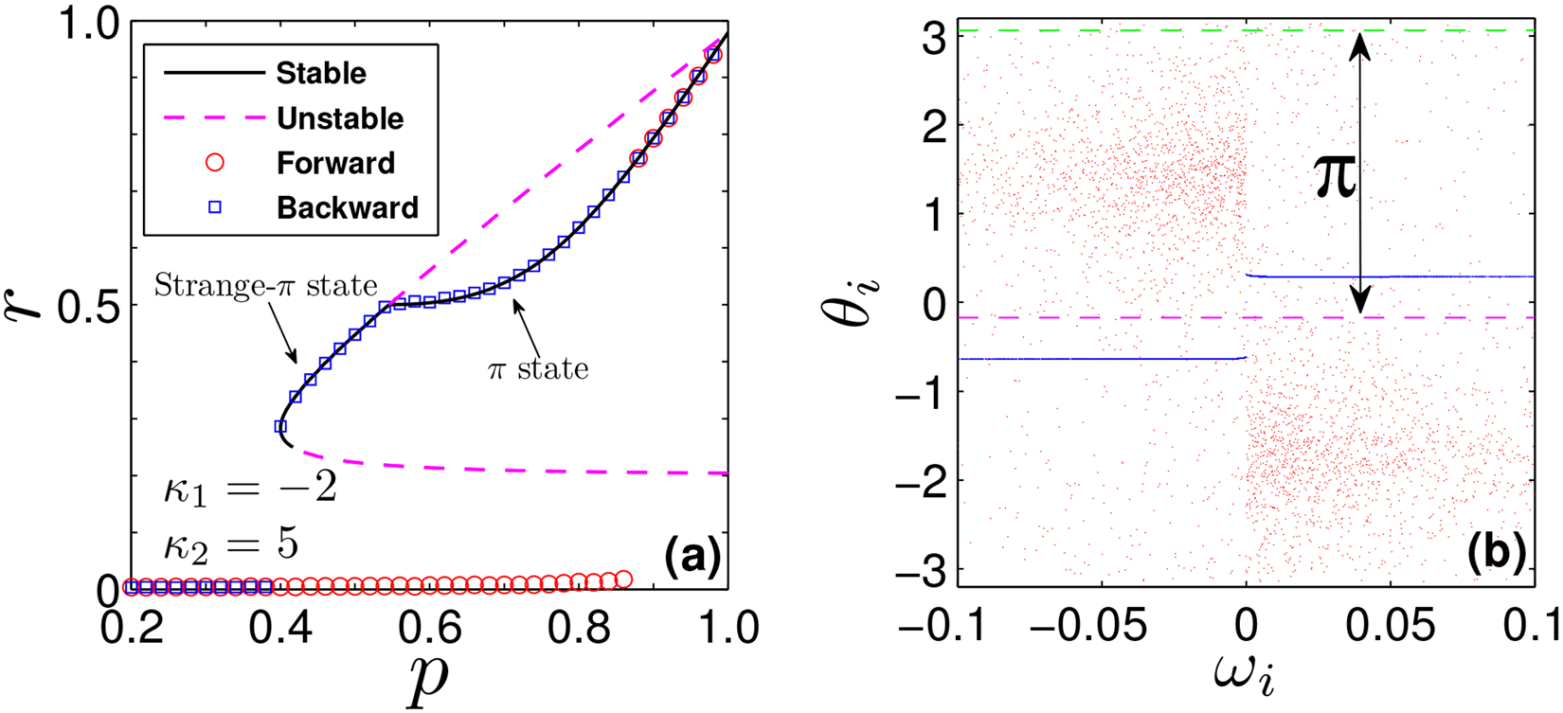
Case I - Strange *π*-state. (**a**) *r* vs. *p*: forward (red circles) and backward (blue squares) transitions are compared with the predicted stable (full black) and unstable (dashed pinkish red) states. (**b**) Snapshot of *θ*_*i*_ vs. *ω*_*i*_, for the strange *π*-state at *p* = 0.5: drifting contrarians (red “clouds”) repel conformists’ clusters (blue lines), keeping to *π* the difference between the average phases of contrarians (dashed green) and conformists (dashed purple).
  With Lorentzian FD, the forward threshold ${p}_{1}^{c}$ (for the transition from incoherence) always exceeds the backward one ${p}_{1}^{b}$ where *π* or strange-*π* states lose their stability. With uniform distributions, instead, one can trigger (by appropriately choosing *κ*_1_) the forward transition so that ${p}_{1}^{c} < {p}_{1}^{b}$. For example, we focus on the case of uniform distribution *g*(*ω*) = 1/2, *ω* ∈ (−1, 1), and |*κ*_1_| = *κ*_2_ = 5. From Eqs (30) and (31), the exact expression for critical proportion of conformists is ${p}_{1}^{c}=(4\sqrt{2}+5\pi )/(10\pi ) < {p}_{1}^{b}=0.7$. In this case, non-stationary states emerge within the interval ${p}_{1}^{c} < {p}_{1} < {p}_{1}^{b}$ with a double hysteresis loop, one near ${p}_{1}^{c}$ and a second one near ${p}_{1}^{b}$ [Fig. 4(a)]. In this regime, oscillators split into four coherent clusters, two for each population. Like *π*-states, different clusters maintains a phase difference of *π* between each other, but oscillators within each clusters are neither phase- nor frequency-locked [Fig. 4(b)]. In fact, they evolve with different periodic patterns [Fig. 4(c)], and correlate with each other so that their average frequencies lock to binary constants ±Ω_*f*_, forming a staircase structure [Fig. 4(b3)]. Henceforth, both populations self-organize their phases as B’s^23,24^, resulting in a macroscopic rhythmic behaviour [Fig. 4(d)]. Numerically, we find that as *p*_1_ increases in the regime from ${p}_{1}^{c}$ to ${p}_{1}^{b}$, the fundamental frequency becomes smaller and smaller, i.e., the period becomes larger and larger. The *π*-state can be consequently be regarded as the limit of a Bellerophon state with infinite period.

**Figure 4:**
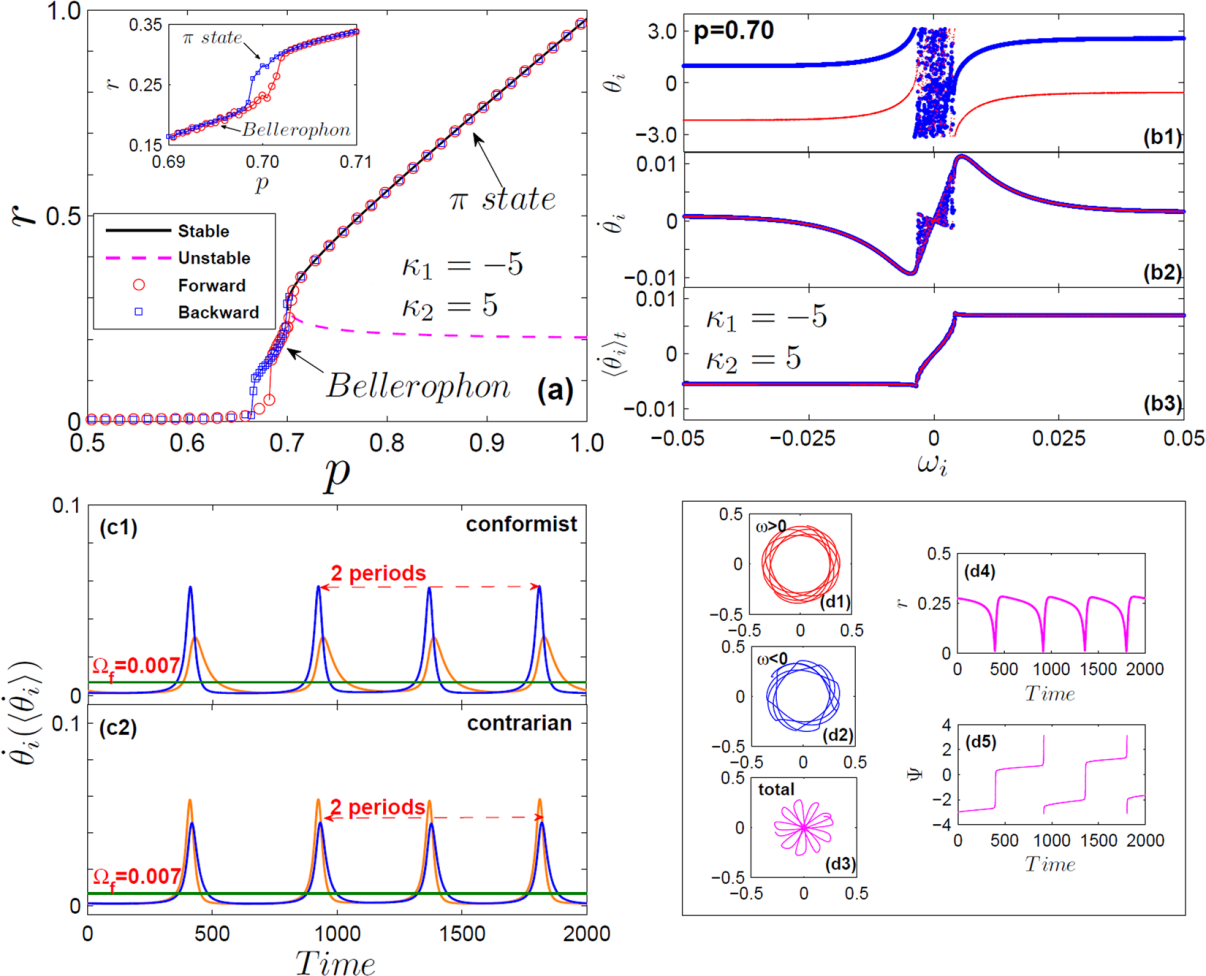
Case I - Bellerophon state. (**a**) *r* vs. *p* for uniform frequency distribution *g*(*ω*) = 1/2, *ω* ∈ (−1, 1), and |*κ*_1_| = *κ*_2_ = 5. Rhythmic states emerge from incoherence (forward) and from the *π*-state (backward) through hysteresis loops, near ${p}_{1}^{c}$ and near ${p}_{1}^{b}$ (see inset). (**b**) Snapshots of the oscillators’ instantaneous phases *θ*_*i*_ (b1), instantaneous frequencies ${\dot{\theta }}_{i}$ (b2), and average frequencies $\langle {\dot{\theta }}_{i}\rangle$ (b3) vs. *ω*_*i*_ (in the B-state at *p* = 0.7) for conformists (blue) and contrarians (red). (**c**) Dynamics of ${\dot{\theta }}_{i}$ for two oscillators of the conformists’ (c1) and the contrarians’ (c2) clusters; straight lines denote their average frequencies locked to the constant Ω_*f*_. (**d**) Left insets (d1–d3): order parameters for oscillators with positive (blue) and negative (red) frequencies, and their average (pinkish red) in the complex plane. Right insets (d4 and d5): corresponding rhythmic evolutions of *r*(*t*) and Ψ(*t*).
2. *Case II*.
  In this case, we substitute Γ_2_(*κ*|*ω*) into Eqs (24) and (25) and obtain
  34 $$\begin{array}{rcl}r & = & -\,{\int }_{-{\omega }_{0}}^{{\omega }_{0}}\,\sqrt{1-{(\frac{\omega -{{\rm{\Omega }}}_{2}}{{\kappa }_{1}\omega r})}^{2}}g(\omega )H(1-|\frac{\omega -{{\rm{\Omega }}}_{2}}{{\kappa }_{1}\omega r}|)d\omega \\  &  & +\,({\int }_{-\infty }^{-{\omega }_{0}}+{\int }_{{\omega }_{0}}^{\infty })\sqrt{1-{(\frac{\omega -{{\rm{\Omega }}}_{2}}{{\kappa }_{2}\omega r})}^{2}}g(\omega )H(1-|\frac{\omega -{{\rm{\Omega }}}_{2}}{{\kappa }_{2}\omega r}|)d\omega ,\end{array}$$
  35 $$\begin{array}{rcl}0 & = & {\int }_{-{\omega }_{0}}^{{\omega }_{0}}\,\frac{\omega -{{\rm{\Omega }}}_{2}}{{\kappa }_{1}|\omega |r}g(\omega )H(1-|\frac{\omega -{{\rm{\Omega }}}_{2}}{{\kappa }_{1}\omega r}|)d\omega \\  &  & +\,({\int }_{-\infty }^{-{\omega }_{0}}+{\int }_{{\omega }_{0}}^{\infty })\frac{\omega -{{\rm{\Omega }}}_{2}}{{\kappa }_{2}|\omega |r}g(\omega )H(1-|\frac{\omega -{{\rm{\Omega }}}_{2}}{{\kappa }_{2}\omega r}|)d\omega \\  &  & +\,{\int }_{-{\omega }_{0}}^{{\omega }_{0}}\,\frac{\omega -{{\rm{\Omega }}}_{2}}{{\kappa }_{1}|\omega |r}[1-\sqrt{1-{(\frac{{\kappa }_{1}\omega r}{\omega -{{\rm{\Omega }}}_{2}})}^{2}}]\,g(\omega )H(|\frac{\omega -{{\rm{\Omega }}}_{2}}{{\kappa }_{1}\omega r}|-1)d\omega \\  &  & +\,({\int }_{-\infty }^{-{\omega }_{0}}+{\int }_{{\omega }_{0}}^{\infty })\frac{\omega -{{\rm{\Omega }}}_{2}}{{\kappa }_{2}|\omega |r}[1-\sqrt{1-{(\frac{{\kappa }_{2}\omega r}{\omega -{{\rm{\Omega }}}_{2}})}^{2}}]\,g(\omega )H(|\frac{\omega -{{\rm{\Omega }}}_{2}}{{\kappa }_{2}\omega r}|-1)d\omega .\end{array}$$
  For partially synchronous state, i.e., |*κ*_1_*r*| > 1 and *κ*_2_*r* > 1, to avoid divergency of Eq. (34), the only choice is Ω_2_ = 0, and Eq. (34) is reduced as
  36 $${p}_{2}=\frac{{r}^{2}+\sqrt{{r}^{2}-{(1/{\kappa }_{1})}^{2}}}{\sqrt{{r}^{2}-{(1/{\kappa }_{1})}^{2}}+\sqrt{{r}^{2}-{(1/{\kappa }_{2})}^{2}}}.$$
  Using the same methods developed in *Case I*, one can identify the entire *π*-state. Interestingly, the result is exactly the same as Eq. (29).
  For the case of |*κ*_1_*r*| < 1 and *κ*_2_*r* < 1, Ω_2_ is not supposed to be 0, the solution of Eqs (34) and (35) can be solved numerically, corresponding to the TW state. Particularly, in the limit case *r* → 0^+^, one can obtain ${p}_{2}^{c}$ again:
  37 $$1={\kappa }_{2}\pi |{{\rm{\Omega }}}_{2}^{c}|g({{\rm{\Omega }}}_{2}^{c})/2,\,{{\rm{\Omega }}}_{2}^{c}\in (\,-\,\infty ,-\,{\omega }_{0}^{c})\cup ({\omega }_{0}^{c},+\,\infty ),$$
  where ${{\rm{\Omega }}}_{2}^{c}$ is the critical mean-field frequency. By Taylor expansion of Eq. (35), one finds that ${{\rm{\Omega }}}_{2}^{c}$ satisfies the following balance equation,
  38 $$0=P.\,V.\,\{\frac{{\kappa }_{1}}{2}\,{\int }_{-{\omega }_{0}}^{{\omega }_{0}}\,\frac{|\omega |g(\omega )}{\omega -{{\rm{\Omega }}}_{2}^{c}}d\omega +\frac{{\kappa }_{2}}{2}\,({\int }_{-\infty }^{-{\omega }_{0}}+{\int }_{{\omega }_{0}}^{\infty })\,\frac{|\omega |g(\omega )}{\omega -{{\rm{\Omega }}}_{2}^{c}}d\omega \}.$$
  Evidently, ${{\rm{\Omega }}}_{2}^{c}$ is the imaginary part of the eigenvalues of operator *A* at the boundary of stability, and ${{\rm{\Omega }}}_{2}^{c}\ne 0$. For a Lorentzian FD, one ultimately obtains
  39 $$\begin{array}{rcl}{{\rm{\Omega }}}_{2}^{c} & = & \pm \tfrac{{\kappa }_{2}+\sqrt{{\kappa }_{2}^{2}-16}}{4}\gamma ,\,{{\rm{\Omega }}}_{2}^{c}\in (\,-\,\infty ,-\,{\omega }_{0}^{c})\cup ({\omega }_{0}^{c},+\,\infty ),\\ {p}_{2}^{c} & = & 1-\tfrac{2}{\pi }\,\arctan \,\sqrt{\tfrac{{z}_{2}-{z}_{2}^{\tfrac{-{\kappa }_{1}}{{\kappa }_{2}-{\kappa }_{1}}}}{1+{z}_{2}^{\tfrac{-{\kappa }_{1}}{{\kappa }_{2}-{\kappa }_{1}}}}},\\ {z}_{2} & = & {({{\rm{\Omega }}}_{2}^{c}/\gamma )}^{2}.\end{array}$$
  As for the TW state, Eqs (34) and (35) can be simplified to
  40 $$\begin{array}{rcl}r & = & -\frac{\gamma }{\pi }\,{\int }_{-\infty }^{\frac{{\omega }_{0}-|{{\rm{\Omega }}}_{2}|}{{\omega }_{0}}}\,\sqrt{1-{(\frac{x}{{\kappa }_{1}r})}^{2}}\frac{|{{\rm{\Omega }}}_{2}|}{{{\rm{\Omega }}}_{2}^{2}+{\gamma }^{2}{(1-x)}^{2}}H\,(1-|\frac{x}{{\kappa }_{1}r}|)\,dx\\  &  & +\,\frac{\gamma }{\pi }\,{\int }_{\frac{{\omega }_{0}-|{{\rm{\Omega }}}_{2}|}{{\omega }_{0}}}^{1}\,\sqrt{1-{(\frac{x}{{\kappa }_{2}r})}^{2}}\frac{|{{\rm{\Omega }}}_{2}|}{{{\rm{\Omega }}}_{2}^{2}+{\gamma }^{2}{(1-x)}^{2}}H\,(1-|\frac{x}{{\kappa }_{2}r}|)\,dx,\end{array}$$
  41 $$\begin{array}{rcl}0 & = & {\int }_{-\infty }^{\frac{{\omega }_{0}-|{{\rm{\Omega }}}_{2}|}{{\omega }_{0}}}\,\frac{x}{{\kappa }_{1}[{{\rm{\Omega }}}_{2}^{2}+{\gamma }^{2}{(1-x)}^{2}]}H\,(1-|\frac{x}{{\kappa }_{1}r}|)\,dx\\  &  & +\,{\int }_{\frac{{\omega }_{0}-|{{\rm{\Omega }}}_{2}|}{{\omega }_{0}}}^{1}\,\frac{x}{{\kappa }_{2}[{{\rm{\Omega }}}_{2}^{2}+{\gamma }^{2}{(1-x)}^{2}]}H\,(1-|\frac{x}{{\kappa }_{2}r}|)\,dx\\  &  & +\,({\int }_{-\infty }^{\frac{{\omega }_{0}-|{{\rm{\Omega }}}_{2}|}{{\omega }_{0}}}-{\int }_{\frac{{\omega }_{0}+|{{\rm{\Omega }}}_{2}|}{{\omega }_{0}}}^{\infty })\,[1-\sqrt{1-{(\frac{{\kappa }_{1}r}{x})}^{2}}]\tfrac{x}{{\kappa }_{1}[{{\rm{\Omega }}}_{2}^{2}+{\gamma }^{2}{(1-x)}^{2}]}H\,(|\frac{x}{{\kappa }_{1}r}|-1)\,dx\\  &  & +\,({\int }_{\frac{{\omega }_{0}-|{{\rm{\Omega }}}_{2}|}{{\omega }_{0}}}^{1}-{\int }_{1}^{\frac{{\omega }_{0}+|{{\rm{\Omega }}}_{2}|}{{\omega }_{0}}})\,[1-\sqrt{1-{(\frac{{\kappa }_{2}r}{x})}^{2}}]\tfrac{x}{{\kappa }_{2}[{{\rm{\Omega }}}_{2}^{2}+{\gamma }^{2}{(1-x)}^{2}]}H\,(|\frac{x}{{\kappa }_{2}r}|-1)\,dx.\end{array}$$
  Numerical results highlight that the bifurcation is supercritical and the TW state is unstable, like the TW state in *Case I*. In Fig. 2(d–f), we have plotted the phase diagram in *Case II*, as well as the solutions of Eq. (36). However, the predictions of *π*-state made by the mean-field theory do not coincide with the numerical results, suggesting that the stationary *π*-state is unstable, which has been further verified by numerical method. In *Case II*, forward thresholds ${p}_{2}^{c}$ smaller than ${p}_{2}^{b}$ commonly appears, leading generically to the emergence of non-stationary coherent states. Typically, the system undergoes two PT’s: one at ${p}_{2}^{c}$, where the incoherent state loses stability and a B-state discontinuously emerges with hysteresis, and another at ${p}_{2}^{c^{\prime} } > {p}_{2}^{c}$, where the Bellerophon state becomes unstable and bifurcates to a novel rhythmic state with a PT whose type changes from discontinuous to continuous as the coupling ratio *Q* ≡ |*κ*_1_|/*κ*_2_ crosses one [Fig. 2(d–f)]. Physically, Q denotes the ratio of entrainment received by the contrarians and conformists from the mean-field.
  In Fig. 5, one such novel rhythmic state is illustrated. In this state, there are six (two pairs of contrarians’ and one pair of conformists’) coherent clusters [Fig. 5(a)]. The pair of contrarian clusters around *ω* = 0 behave like odd B’s [reflected by the plateaus in panel (a3)] and rotate over all the unit circle [panel (b1)]; the other two pairs of clusters (with larger |*ω*|) behave instead as even B’s (a.k.a. oscillating *π*-states^24^) oscillating like shuttle-run in limited arches [panel (b2)]. Note that their average frequencies are zero [panel (a3)]. Being a superposition of odd and even B’s, we refer to such a macroscopic rhythmic state as hybrid-Bellerophon state.

**Figure 5:**
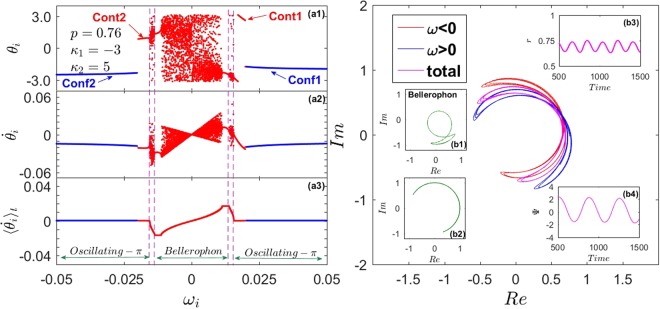
Case II - Hybrid-Bellerophon states. (**a**) Microscopic features of the hybrid-B’s at *p* = 0.76 in the forward PT reported in Fig. 2(d): distribution of *θ*_*i*_ (a1), ${\dot{\theta }}_{i}$ (a2), and $\langle {\dot{\theta }}_{i}\rangle$ (a3) vs. *ω*_*i*_. One can identify six (two conformist and four contrarian) coherent clusters of oscillators, whose phases are self-organized as in pure B’s and in oscillating *π*-states. (**b**) The order parameters for all oscillators with positive (blue) and negative (red) frequencies, and their frequency average (pinkish red). The insets plot the typical rhythmic behaviors of the order parameter restricted to the clusters of oscillators in B (b1) and in oscillating-*π* (b2) states. Panels (b3 and b4) report the rhythmic evolution in time of the global order parameters *r*(*t*) and Ψ(*t*), respectively.
  To conclude, there are two synchronization PT’s in *Case II*. Further numerical simulations show that when *κ*_2_ is a constant, with the increasing of |*κ*_1_|, the width of hysteresis of the first PT almost remains the same, but the width of hysteresis of the second PT becomes smaller and smaller. When *Q* = |*κ*_1_|/*κ*_2_ < 1, the two staircases of Bellerophon state are one in the forward PT and the other in the backward PT. When *Q* = |*κ*_1_|/*κ*_2_ = 1, the two staircases start joining with each other. Until *Q* = |*κ*_1_|/*κ*_2_ > 1, the two staircases coincides with each other and the hysteresis disappears eventually. Although the second PT becomes a continuous one eventually, the hysteresis of the first PT is still observable. We can conclude that the bifurcation of the Bellerophon state is always subcritical, although the hybrid-Bellerophon state will emerge from subcritical bifurcation to supercritical bifurcation with the increasing of |*κ*_1_|.
3. *Case III*.

Substituting Γ_3_(*κ*|*ω*) into Eqs (24) and (25), one obtains

42 $$\begin{array}{rcl}r & = & {\int }_{-{\omega }_{0}}^{{\omega }_{0}}\,\sqrt{1-{(\frac{\omega -{{\rm{\Omega }}}_{3}}{{\kappa }_{2}\omega r})}^{2}}g(\omega )H\,(1-|\frac{\omega -{{\rm{\Omega }}}_{3}}{{\kappa }_{2}\omega r}|)\,d\omega \\  &  & -\,({\int }_{-\infty }^{-{\omega }_{0}}+{\int }_{{\omega }_{0}}^{\infty })\sqrt{1-{(\frac{\omega -{{\rm{\Omega }}}_{3}}{{\kappa }_{1}\omega r})}^{2}}g(\omega )H\,(1-|\frac{\omega -{{\rm{\Omega }}}_{3}}{{\kappa }_{1}\omega r}|)\,d\omega ,\end{array}$$

43 $$\begin{array}{rcl}0 & = & {\int }_{-{\omega }_{0}}^{{\omega }_{0}}\,\frac{\omega -{{\rm{\Omega }}}_{3}}{{\kappa }_{2}|\omega |r}g(\omega )H\,(1-|\frac{\omega -{{\rm{\Omega }}}_{3}}{{\kappa }_{2}\omega r}|)\,d\omega \\  &  & +\,({\int }_{-\infty }^{-{\omega }_{0}}+{\int }_{{\omega }_{0}}^{\infty })\,\frac{\omega -{{\rm{\Omega }}}_{3}}{{\kappa }_{1}|\omega |r}g(\omega )H\,(1-|\frac{\omega -{{\rm{\Omega }}}_{3}}{{\kappa }_{1}\omega r}|)\,d\omega \\  &  & +\,{\int }_{-{\omega }_{0}}^{{\omega }_{0}}\,\frac{\omega -{{\rm{\Omega }}}_{3}}{{\kappa }_{2}|\omega |r}[1-\sqrt{1-{(\frac{{\kappa }_{2}\omega r}{\omega -{{\rm{\Omega }}}_{3}})}^{2}}]\,g(\omega )H\,(|\frac{\omega -{{\rm{\Omega }}}_{3}}{{\kappa }_{2}\omega r}|-1)\,d\omega \\  &  & +\,({\int }_{-\infty }^{-{\omega }_{0}}+{\int }_{{\omega }_{0}}^{\infty })\frac{\omega -{{\rm{\Omega }}}_{3}}{{\kappa }_{1}|\omega |r}[1-\sqrt{1-{(\frac{{\kappa }_{1}\omega r}{\omega -{{\rm{\Omega }}}_{3}})}^{2}}]\,g(\omega )H\,(|\frac{\omega -{{\rm{\Omega }}}_{3}}{{\kappa }_{1}\omega r}|-1)\,d\omega .\end{array}$$

For the partially synchronous state, i.e., |*κ*_1_*r*| > 1 and *κ*_2_*r* > 1, to avoid divergency of Eq. (42), the only choice is Ω_3_ = 0, and Eq. (42) can be reduced as

44 $${p}_{3}=\frac{{r}^{2}+\sqrt{{r}^{2}-{(1/{\kappa }_{1})}^{2}}}{\sqrt{{r}^{2}-{(1/{\kappa }_{1})}^{2}}+\sqrt{{r}^{2}-{(1/{\kappa }_{2})}^{2}}}.$$

From Eq. (44), one can obtain the entire *π*-state, as well as extract the critical proportion of conformists where the *π*-state loses its stability in backward PT (${p}_{3}^{b}$). Interestingly, it is exactly the same as Eq. (29) for *Case I* and Eq. (36) for *Case II*. The solutions of Eq. (44) are plotted in Fig. 1(c). Remarkably, unlike the case of Γ_2_(*κ*|*ω*), the predictions of *π*-state made by the mean-field theory coincide perfectly with the numerical data. This suggests that the mean-field theory still holds in this case, and the stationary *π*-state is a stable synchronous state. To sum up, the theoretical predictions of stationary *π*-state are identical in all cases, only in *Case II* it is unstable where a non-stationary strange *π*-state replaces it.

For the case of |*κ*_1_*r*| < 1 and *κ*_2_*r* < 1, Ω_3_ is not supposed to be 0, the solution of Eqs (42) and (43) can be solved numerically, corresponding to the TW state. Particularly, in the limit case *r* → 0^+^, one can obtain ${p}_{3}^{c}$:

45 $$1=\frac{{\kappa }_{2}\pi |{{\rm{\Omega }}}_{3}^{c}|g({{\rm{\Omega }}}_{3}^{c})}{2},\,{{\rm{\Omega }}}_{3}^{c}\in (\,-\,{\omega }_{0}^{c},{\omega }_{0}^{c}),$$

where ${{\rm{\Omega }}}_{3}^{c}$ is the critical mean-field frequency. By Taylor expansion of Eq. (43), ${{\rm{\Omega }}}_{3}^{c}$ satisfies

46 $$0=P.V.\,\{\frac{{\kappa }_{2}}{2}\,{\int }_{-{\omega }_{0}}^{{\omega }_{0}}\,\frac{|\omega |g(\omega )}{\omega -{{\rm{\Omega }}}_{3}^{c}}d\omega +\frac{{\kappa }_{1}}{2}\,({\int }_{-\infty }^{-{\omega }_{0}}+{\int }_{{\omega }_{0}}^{\infty })\,\frac{|\omega |g(\omega )}{\omega -{{\rm{\Omega }}}_{3}^{c}}\,d\omega \}.$$

${{\rm{\Omega }}}_{3}^{c}$ is also the imaginary part of the eigenvalues of operator *A* at the boundary of stability, and ${{\rm{\Omega }}}_{3}^{c}\ne 0$. For a Lorentzian FD, one ultimately obtains ${{\rm{\Omega }}}_{3}^{c}=\pm \,\gamma ({\kappa }_{2}-\sqrt{{\kappa }_{2}^{2}-16})/4$, ${{\rm{\Omega }}}_{3}^{c}\in (\,-\,{\omega }_{0}^{c},{\omega }_{0}^{c})$, and ${p}_{3}^{c}=\frac{2}{\pi }\,\arctan \,\sqrt{({z}_{3}+{z}_{3}^{\tfrac{{\kappa }_{2}}{{\kappa }_{2}-{\kappa }_{1}}})/(1-{z}_{3}^{\tfrac{{\kappa }_{2}}{{\kappa }_{2}-{\kappa }_{1}}})}$, ${z}_{3}={({{\rm{\Omega }}}_{3}^{c}/\gamma )}^{2}$.

As for the TW state, Eqs (42) and (43) can be simplified to

47 $$\begin{array}{rcl}r & = & \frac{\gamma }{\pi }\,{\int }_{-\infty }^{\frac{{\omega }_{0}-|{{\rm{\Omega }}}_{3}|}{{\omega }_{0}}}\,\sqrt{1-{(\frac{x}{{\kappa }_{2}r})}^{2}}\frac{|{{\rm{\Omega }}}_{3}|}{{{\rm{\Omega }}}_{3}^{2}+{\gamma }^{2}{(1-x)}^{2}}H\,(1-|\frac{x}{{\kappa }_{2}r}|)\,dx\\  &  & -\,\frac{\gamma }{\pi }\,{\int }_{\frac{{\omega }_{0}-|{{\rm{\Omega }}}_{3}|}{{\omega }_{0}}}^{1}\,\sqrt{1-{(\frac{x}{{\kappa }_{1}r})}^{2}}\frac{|{{\rm{\Omega }}}_{3}|}{{{\rm{\Omega }}}_{3}^{2}+{\gamma }^{2}{(1-x)}^{2}}H\,(1-|\frac{x}{{\kappa }_{1}r}|)\,dx,\end{array}$$

48 $$\begin{array}{rcl}0 & = & {\int }_{-\infty }^{\frac{{\omega }_{0}-|{{\rm{\Omega }}}_{3}|}{{\omega }_{0}}}\,\frac{x}{{\kappa }_{2}[{{\rm{\Omega }}}_{3}^{2}+{\gamma }^{2}{(1-x)}^{2}]}H\,(1-|\frac{x}{{\kappa }_{2}r}|)\,dx\\  &  & +\,{\int }_{\frac{{\omega }_{0}-|{{\rm{\Omega }}}_{3}|}{{\omega }_{0}}}^{1}\,\frac{x}{{\kappa }_{1}[{{\rm{\Omega }}}_{3}^{2}+{\gamma }^{2}{(1-x)}^{2}]}H\,(1-|\frac{x}{{\kappa }_{1}r}|)\,dx\\  &  & +\,({\int }_{-\infty }^{\frac{{\omega }_{0}-|{{\rm{\Omega }}}_{3}|}{{\omega }_{0}}}-{\int }_{\frac{{\omega }_{0}+|{{\rm{\Omega }}}_{3}|}{{\omega }_{0}}}^{\infty })\,[1-\sqrt{1-{(\frac{{\kappa }_{2}r}{x})}^{2}}]\tfrac{x}{{\kappa }_{2}[{{\rm{\Omega }}}_{3}^{2}+{\gamma }^{2}{(1-x)}^{2}]}H\,(|\frac{x}{{\kappa }_{2}r}|-1)\,dx\\  &  & +\,({\int }_{\frac{{\omega }_{0}-|{{\rm{\Omega }}}_{3}|}{{\omega }_{0}}}^{1}-{\int }_{1}^{\frac{{\omega }_{0}+|{{\rm{\Omega }}}_{3}|}{{\omega }_{0}}})\,[1-\sqrt{1-{(\frac{{\kappa }_{1}r}{x})}^{2}}]\tfrac{x}{{\kappa }_{1}[{{\rm{\Omega }}}_{3}^{2}+{\gamma }^{2}{(1-x)}^{2}]}H\,(|\frac{x}{{\kappa }_{1}r}|-1)\,dx.\end{array}$$

**Figure 6:**
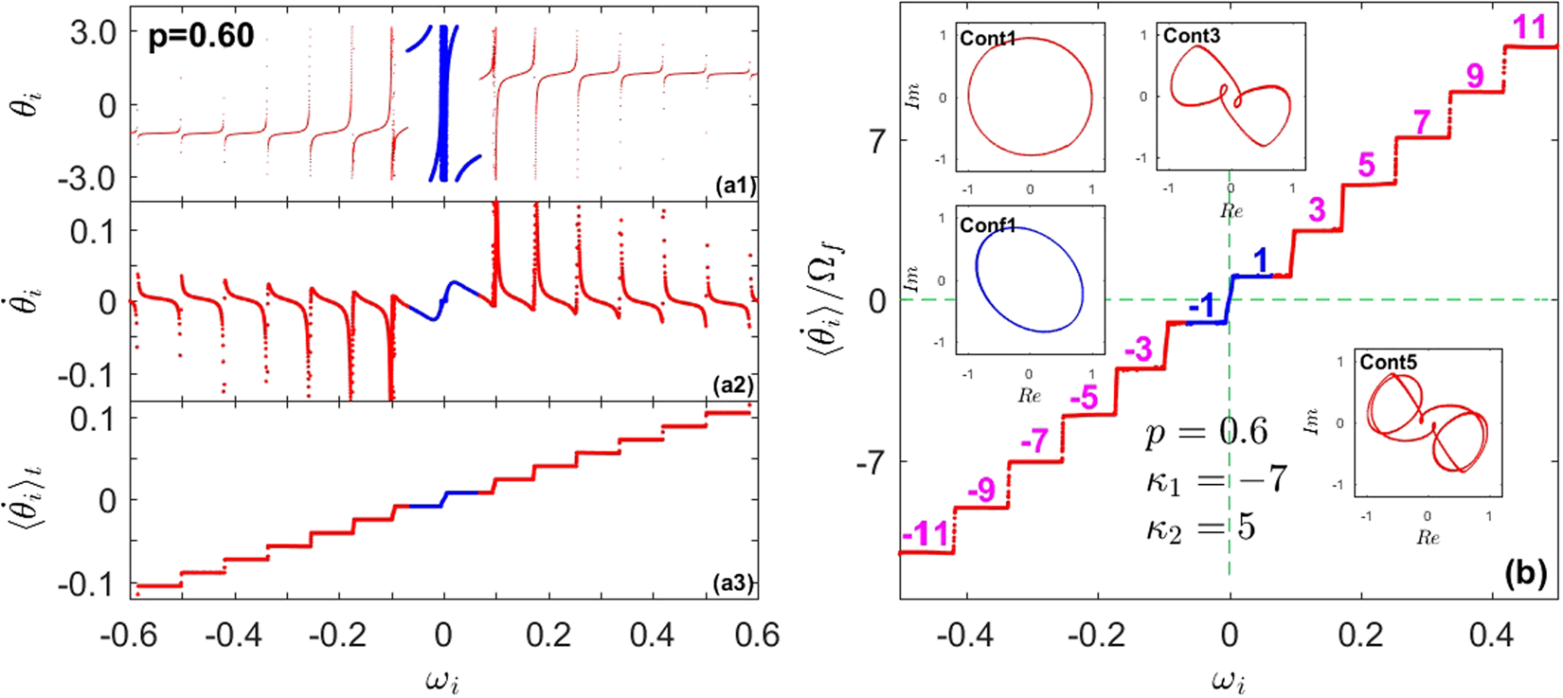
Case III - B-state with quantized clusters. (**a**) Microscopic features of the B-state at *p* = 0.6 in the forward PT reported in Fig. 2(i): distribution of *θ*_*i*_ (a1), ${\dot{\theta }}_{i}$ (a2), and $\langle {\dot{\theta }}_{i}\rangle$ (a3) vs. *ω*_*i*_. Conformists and contrarians are represented in blue and red, respectively. (**b**) Distribution of the average frequencies in units of the fundamental frequency Ω_*f*_ , unveiling the odd-multiplicity rule $\langle {\dot{\theta }}_{i}\rangle /{{\rm{\Omega }}}_{f}=n$ with *n* = 1, 3, 5, … behind the staircase structure in (a3). The insets plot the order parameters for the coherent clusters of conformist (*Conf*(±1)) and contrarians (*Cont*(±*n*), with *n* = 1, 3, 5) in the complex plane.

The solutions of Eqs (47) and (48) are reported in Fig. 2(g–i), and numerical results give evidence that this bifurcation can be stable only in the case of *κ*_2_ > |*κ*_1_|. The theoretical predictions agree perfectly with the numerical results. Depending on *κ*, one finds three main processes of synchronization. When *Q* < 1, ${p}_{3}^{c} > {p}_{3}^{b}$, and a stable *π*-state emerge discontinuously out of incoherence near ${p}_{3}^{c}$ [Fig. 2(g)]. When *Q* = 1, ${p}_{3}^{c} < {p}_{3}^{b}$, the incoherent state bifurcates discontinuously towards a B-state with hysteresis near ${p}_{3}^{c}$, and then the B-state loses stability at ${p}_{3}^{b}$ towards a *π*-state with a continuous transition [Fig. 2(h)]. Finally, when *Q* > 1, again ${p}_{3}^{c} < {p}_{3}^{b}$ and the results regarding the first transition are the same as *Q* = 1, with the main exception that here a stable traveling wave (TW) state emerges continuously from the Bellerophon state and it vanishes at ${p}_{3}^{c^{\prime} } > {p}_{3}^{b}$ with a first-order-like transition to the *π*-state, resulting overall in a three-stage PT to synchronization [Fig. 2(i)]. Unlike the previous cases, here both populations in the Bellerophon state self-organize their phases by splitting into multiple coherent clusters separated by “seas” of drifting oscillators [Fig. 6(a)]. Within each coherent cluster, the oscillators’ average frequencies (the ensemble averaged frequency^39^) are locked to odd multiple integers of a fundamental (lowest) frequency Ω_*f*_^23,24^, so that clusters form a staircase structure described by Ω_±*n*_ = ±(2*n* − 1)Ω_*f*_ with $n\in {\mathbb{N}}$ [Fig. 6(b)]. Due to the correlation among oscillators in the clusters, the order parameter exhibits complicated orbits in the complex plane, as shown in the insets of Fig. 6(b).

To summarize, there are three different processes of synchronization depending on the relative magnitude of |*κ*_1_| and *κ*_2_ in *Case III*. When *Q* < 1, ${p}_{3}^{c} > {p}_{3}^{b}$. As the incoherent state loses its stability at ${p}_{3}^{c}$, the stationary TW solution predicted by Eqs (47) and (48) is unstable, thus the *π*-state predicted by Eq. (44) emerges with a hysteresis near ${p}_{3}^{c}$. As the proportion of conformists increases, the system remains *π*-state afterwards. Therefore, in this synchronization process there is only one first-order PT from incoherent state to *π*-state. When *Q* = 1, ${p}_{3}^{c} < {p}_{3}^{b}$. As the incoherent state loses its stability at ${p}_{3}^{c}$, a non-stationary Bellerophon state emerges. The bifurcation of this Bellerophon state is subcritical, thus non-stationary *r*(*t*) emerges with a hysteresis near ${p}_{3}^{c}$. As *p*_3_ increases, it eventually vanishes at ${p}_{3}^{b}$ with a continuous transition to the *π*-state. Therefore, in this synchronization process there are two-stage synchronization PTs, one is a discontinuous PT from incoherent state to the Bellerophon state, and the other is a continuous PT from the Bellerophon state to *π*-state. When *Q* > 1, the results turn out to be the same as *Q* = 1, except that the TW state can be stable and it vanishes at ${p}_{3}^{c^{\prime} } > {p}_{3}^{b}$ with a discontinuous transition to the *π*-state as *p*_3_ increases. Further numerical simulations show that if |*κ*_1_| is large enough, the Bellerophon state will emerge from subcritical bifurcation to supercritical bifurcation, which causes the first PT change from a discontinuous one to a continuous one. In conclusion, with the increasing of |*κ*_1_|, the first PT changes from first-order to second-order, meanwhile the second PT emerges and changes from the second-order to the first-order.

## Discussion

While in the classical Kuramoto model, phase oscillators are globally and homogeneously coupled to the mean field, in some practical situations such a coupling may instead be heterogeneous. In ref.^23^, a frequency-weighted Kuramoto model was studied, and a novel quantized, time-dependent, and clustered state (the Bellerophon state) was revealed. In ref.^24^, Bellerophon states were again observed in coupled conformist and contrarian oscillators.

Here, we combined frequency-weighted coupling strengths with positive and negative interactions. As compared with refs^23,24^, such a higher order of heterogeneity gives rise to a much richer scenario, which includes different regions of bi-stability. Furthermore, we identified the multiple phase transition character of the root to synchronization, and gave evidence of the generic emergence of macroscopic rhythmic regimes where oscillators self-organize as in Bellerophon states. Together with the Bellerophon states of refs^23,24^, novel collective phases (namely, the strange *π*- and the hybrid B-states) were revealed. Unlike precedent studies, where periodic synchronization behaviors were observed in the presence of an external periodic driving^40–42^, the collective rhythms here reported emerge spontaneously, as soon as the forward critical threshold precedes the backward one.

The strange *π*- and hybrid B-states observed in the present model can be regarded as a transitional state between the incoherent state (full asynchrony) and the *π*-state (full synchrony). On the one hand, the control parameter is not strong enough to completely entrain the system into the *π*-state, on the other hand it is large enough to maintain certain correlations among the instantaneous frequencies of oscillators. As a compromise of this competition, the instantaneous frequencies of oscillators are not locked but their average frequencies are locked to certain constant.

The current model of conformist and contrarian may describe neuron systems, political systems, or economical systems. The rich synchronization phenomena and the nontrivial coherent states observed in this work could help us better understand the collective behaviors in such systems. For instance, diverse neuro-degenerative disorders, like Parkinson’s disease, have been proved to be correlated to the spontaneous emergence of global neuronal oscillations, which makes the present model appealing for a better understanding of their dynamical origins. Moreover, due to their ubiquitous appearance, our study paves the possibility that the microscopic, quantized features of B’s are actually the fundamental building blocks behind spontaneous emergence of collective rhythms in more general systems of interacting oscillators, opening a theoretical challenge in the study of rhythmic synchronization.
